# Reinforcement effect of intra-orifice barrier materials in teeth treated with regenerative endodontic procedure: Research article

**DOI:** 10.34172/joddd.2021.019

**Published:** 2021-05-05

**Authors:** Sevinç Aktemur Türker, Keziban Olcay, Sena Kaşıkçı, Fatma Zühal Yurdagül

**Affiliations:** ^1^Department of Endodontics, Faculty of Dentistry, Zonguldak Bülent Ecevit University, Zonguldak,Turkey.; ^2^Department of Endodontics, Faculty of Dentistry, İstanbul University-Cerrahpaşa, İstanbul,Turkey.

**Keywords:** Immature teeth, Intra-orifice barrier material, ProRoot MTA, Regenerative endodontic procedure, Root fracture

## Abstract

**Background.** Regenerative endodontic treatment (RET) is a clinically advanced procedure for necrotic immature teeth. However, root canal walls of these teeth are brittle especially in the cervical region and need reinforcement. This *in vitro* study is conducted to evaluate the effect of intra-orifice barrier materials on the fracture resistance of immature teeth treated with regenerative procedure.

**Methods.** Forty-eight maxillary central incisors were used. Twelve intact teeth were selected for the control group. Remained teeth were prepared using peeso drills to simulate immature teeth and assigned into three groups according to the intra-orifice barrier material placed over MTA (n = 12); Composite resin (CR), ProRoot MTA and Resin-modified glass ionomer cement (RMGIC). Fracture strength test was applied using a universal testing machine. One-way ANOVA and Tukey post hoc tests were used at *P* = 0.05.

**Results.** A significant difference was obtained among groups (*P* < 0.05). MTA showed the lowest fracture resistance (*P* < 0.05). However, no significant difference was found among RMGIC, CR, and control groups (*P* > 0.05).

**Conclusion.** Intra-orifice restorative materials have reinforcement affect in immature teeth treated with regenerative endodontic procedure. RMGIC or CR can be regarded as a viable choice to reduce the occurrence of cervical root fracture of immature teeth treated with a regenerative therapy.

## Introduction

Regenerative endodontic treatment (RET) has been applied as an alternative treatment procedure to apexification for open apex teeth with necrotic pulp and apical periodontitis.^1^ This treatment aims to provide further root development and strengthen the root. However, even with RET, the cervical region does not develop further.^2^ These teeth are shown more susceptible to cervical fracture during functional stresses and secondary to trauma applied to the cervical area.^3,4^ In RET, cervical sealing with a tricalcium silicate-based barrier is suggested to provide a bacterial tight seal^5^ and induction of mineral formation.^6^ Composites, in combination with dental adhesives, have been commonly suggested for an effective coronal restoration placed over the tricalcium silicate-based barrier to prevent reinfection of the root canal.^7^ To date, several case reports related to teeth treated with a RET protocol have been published. In these reports, commonly, the tricalcium silicate-based barrier was covered solely with composite resin restorations to seal the access cavity. According to the recent case reports, it was demonstrated that cervical fracture was the prime cause of failure in open apex teeth treated with RET followed by a coronal composite restoration.^8-10^ Arslan et al^8^ reported a horizontal crown fracture that occurred three years, five months after RET. Similarly, Shimizu et al^9^ and Martin et al^10^ demonstrated crown fractures approximately two years after the completion of the RET. At that point, it can be concluded that although RET is a clinically advanced procedure, root canal walls in the cervical area remain brittle and need reinforcement.^11^

The use of intra-orifice barriers was shown to reinforce endodontically treated teeth against root fracture.^12^ To date, to the authors’ knowledge, the reinforcing effect of intra-orifice restorative materials placed over MTA in RET has not been assessed. Therefore, this study aimed to evaluate the reinforcing effect of three intra-orifice barrier materials (composite resin [CR], and ProRoot MTA and resin-modified glass ionomer cement [RMGIC]) in immature teeth treated with regenerative procedure. The null hypothesis tested was that there would be no difference in the reinforcing effects of intra-orifice barrier materials.

## Methods

Forty-eight human maxillary incisor teeth, selected from a random collection of extracted teeth unrelated with this study, which were stored in a 0.9% physiologic saline with 0.1% thymol solution, were used. The sample size was calculated as 12 in each group, with 0.35 effect sizes, a type I error of 0.05, and a statistical power of 80% using the G*Power software (version 3.1.9.7). All procedures performed in studies were in accordance with the ethical standards of the institutional national research committee and with the 1964 Helsinki declaration and its later amendments or comparable ethical standards. The mesiodistal and buccolingual diameters of the teeth were measured and teeth with similar sizes were used. The crowns were partially decoronated to obtain a standard length to 20 mm. Twelve intact teeth were assigned as control group.

Access cavities were prepared in the remaining 36 teeth and the root canals were prepared from coronal to apical direction with peeso drills between #1 and #5 at 1 mm beyond the apex. Thereafter, a size 6 peeso reamer was used to extend the preparation of the canal 3 mm below the cementoenamel junction to approximate Cvek’s stage 3 of root development as described by Cicek et al^13^ 2.5% NaOCl was used during preparation of root canals. For final irrigation root canals were irrigated with 5 mL of 17% EDTA and 10 mL of distilled water and then dried with paper points.

**Figure 1:**
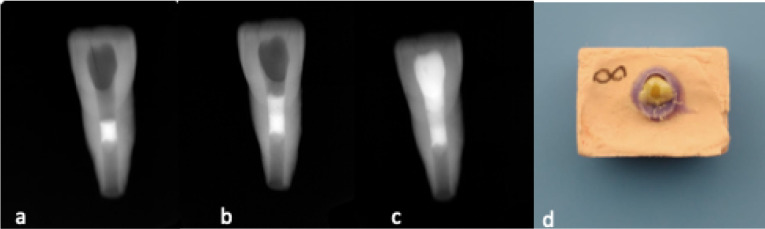
Representative images for placement of (a) 3mm of MTA (b) 2mm of an intra-orifice restorative barrier material (c) composite resin and (d) a fractured sample.

ProRoot MTA (Dentsply Maillefer, Ballaigues, Switzerland) was mixed according to the manufacturer’s instructions. The thickness of MTA was obtained using customized gutta-percha points, which were inserted into the root canal from apical to coronal direction as described in a previous report by Küçükkaya Eren et al.^14^ A gutta-percha point that fits tightly in the root canal was shortened to the appropriate length for each specimen and then MTA was placed from coronal access 2 mm below the cement-enamel junction (with 3-mm thickness) with an MTA carrier (Medesy, Maniago, Italy) (Figure 1a). After 2 hours, 45 min of incubation for MTA setting, 36 teeth were randomly divvied into three groups (n=12) according to the intra-orifice barrier material placed over MTA (Figure 1b).

Group RMGIC: GC Fuji II LC light-cured reinforced glass ionomer cement capsule (GC, America Inc. Alsip, IL, USA) was placed into an amalgamator and mixed for 10 seconds according to manufacturer’s introductions. GC cavity conditioner was applied to the intra-orifice of the root canal. This conditioner was rinsed away. Then 2-mm of RMGIC placed inside the orifice over the MTA and polymerized for 20 seconds with a light-curing device.

Group CR: After the use of a one-step self-etching adhesive, OptiBond^TM^ All-In-One (Kerr, Orange, CA, USA) a 2 mm of composite resin (Point 4, Kerr, Orange, CA, USA) was applied over MTA by hand plugger and polymerized as described above.

Group MTA: ProRoot MTA was mixed in a 3:1 powder to liquid ratio based on the manufacturer’s instructions and 2-mm of MTA placed inside the orifice over the MTA. Then, wet cotton was placed on MTA for about 2 hours 45 minutes until it was completely hardened.

In all groups a final composite resin (Point 4, Kerr, Orange, CA, USA) restoration was placed in increments of 2 mm to the access cavity according to the manufacturer’s instructions and polymerized for 20 seconds (Figure 1c).

### Fracture strength test

To simulate the periodontal membrane, a thin layer of polyvinyl siloxane impression material was applied to the root surfaces. Then roots were mounted vertically 2-mm below the cement-enamel junction in self-cure acrylic resin blocks. Blocks were placed in a universal testing machine (Shimadzu, Kyoto, Japan) and a compressive loading at a speed of 0.5 mm/min was applied the cingulum at a 135° angle with a spherical tip.^3^ The force when the fracture occurred was calculated in Newton (Figure 1d).

### Statistical analysis

The statistical analysis was performed using SPSS 20 (IBM SPSS Inc., Chicago, IL, USA). Normality of the data was tested with Kolmogorov-Smirnov test. Data were analyzed with the one-way analysis of variance (ANOVA) and Tukey post hoc tests at *P* = 0.05.

## Results

Table 1 shows the results of the current study. A significant difference was obtained among groups (*P* = 0.024). MTA showed the lowest fracture resistance (*P* = 0.025). The control group presented the highest values, followed by CR group and RMGIC group, respectively. However, no significant difference was found among RMGIC, CR, and control groups (*P* = 0.875).

**Table 1:** Mean and standard error of fracture strength in the experimental and control groups in newton (N)

**Groups**	**Mean**	**Standard error**
Control	915.68^a^	197.41
RMGIC	805.13^a^	59.44
CR	909.20^a^	101.46
MTA	522.52^b^	35.57

Different superscript letters represent statistically significant differences (*P* < 0.05) in the same column.

## Discussion

This study was conducted to compare the reinforcement effect of three intra-orifice barrier materials in teeth treated with RET. RMGIC, MTA, and CR were used as an intra-orifice barrier placed over MTA. To the best of authors’ knowledge, this is the first study using these restorative materials at the intra-orifice of teeth treated with RET and comparing their reinforcing effect.

Due to the lack of any studies on this topic, the results of the present study were compared with *in vitro* studies that investigated the effect of intra-orifice barrier materials on the fracture resistance of root-filled teeth.^12,15-17^ According to the results the reinforcing effect of the intra-orifice barrier materials was statistically significant. Intact teeth with higher dentin thickness at the cervical area (control group) were showed the highest fracture resistance values. Root reinforcement with RMGIC or CR intra-orifice barriers reduced the susceptibility of roots to root fracture with no significant difference compared to control teeth. However, MTA did not strengthen the root. These results are consonant with previous reports.^12,17^ Nagas et al^12^ and Gupta et al^17^ reported that RMGIC and fiber-reinforced composite materials improved the fracture resistance when used as intra-orifice barriers, whereas MTA did not exhibit any reinforcing effect. Low fracture resistance of MTA may be attributed to its lack of bonding to the dentin, high stiffness in compression, and little strength in tension.^17^ Huang et al^18^ reported that a mineral-rich, collagen degradation zone with reduced flexural strength is shown after applying MTA. This was attributed to destroying the collagen by the hydroxide ions which are capable of infiltrating mineralized collagen. In another study by Nagas et al,^15^ it was demonstrated that a fiber-incorporated version of MTA could significantly contribute to higher fracture resistance values. Conversely, in a study by Savadi Oskoee et al^16^ no significant difference was found between MTA and RMGIC. The disagreements in the findings among studies may be attributed to the differences in study designs.

To reinforce the cervical region of immature teeth the use of composite resin has been recommended.^19^ Composite resins bond to the tooth structure micro mechanically, reportedly absorb and distribute forces in a uniform manner, thereby increasing resistance to fracture and providing an improved prognosis.^17^ It can be concluded that, when a composite resin is used as an intra-orifice barrier and followed by a coronal composite restoration, a single entity is formed at the cervical area of the root and as a result, the forces are absorbed and better distributed in a uniform manner.

RMGICs contain some methacrylate components standard in resin composites and have been used as an acceptable coronal seal.^20^ According to results of this study, RMGIC showed similar reinforcing effect as compared to composite and the control group. To strengthen the roots, materials with a modulus of elasticity similar to that of dentin should be prefer to minimize the stress concentrations at the dentin-material interface.^21^ Due to the closeryoung’s modulus of RMGIC (10-14 GPa) to that of dentin, RMGIC can stand up to a large amount of load before transmitting to the root.^22^

## Conclusion

Reinforcement of immature teeth treated with RMGIC or CR as intra-orifice barriers can be regarded as a viable choice to reduce the occurrence of cervical root fracture. Further investigations especially clinical trials should be performed to evaluate these effects to obtain information that could be extrapolated to clinical practice.

## Authors’ Contributions

Conception or design of the work done by SAT, KO. The acquisition, analysis, or interpretation of data for the work done by SK, FZY and KO.Drafting the work or revising it critically for important intellectual content done by SAT and KO.

## Funding

This study was not supported by any fund source.

## Competing Interests

The authors are declaring that there are no financial and non-financial competing interests with regards to the publication of this work.

## Ethics approval

All procedures performed in studies involving human participants were in accordance with the ethical standards of the institutional and/or national research committee and with the 1964 Helsinki declaration and its later amendments or comparable ethical standards. Verbal informed consent was obtained from all individual participants included in the study.
